# The most unkindest cut

**DOI:** 10.15252/embr.202154200

**Published:** 2021-11-05

**Authors:** Howy Jacobs

**Affiliations:** ^1^ Tampere University Tampere Finland

## Abstract

When universities need to make staffing cuts to balance the books, do they always do so in the fairest and most rational manner?

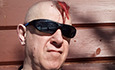

Universities, like most other large organizations, undergo periodic restructuring, expansions and contractions, driven largely by changes in their balance sheet. The contractions are often abrupt and painful. Typically, this outcome is preceded by a period when the top management already perceived a growing problem but postponed action because of its debilitating effects on morale and on the organization’s core functions, in the hope that “something might turn up” that would obviate the need for drastic cuts. Most often, something does not turn up, and the cuts end up being even more severe.

The first sign of trouble is usually a “message to all staff” that seems to come out of the blue, couched in the most anodyne of terms, or even trying to put a positive spin on what is a fundamentally destructive process. But staff about to be made redundant do not appreciate being referred to as “person‐years”. It also seems completely pointless to dress up more or less arbitrary decisions on whom to terminate as “negotiations”, thus apportioning a share of blame to the union representatives who have not much say in these “negotiations” anyway. Although the cuts typically affect non‐academic staff, such as lab technicians, IT and audiovisual support, financial administrators, providers of student welfare services or travel and hospitality arrangers, academic staff at all levels are sometimes affected as well.

Most of us can read between the lines and find disingenuous statements of this type insulting rather than reassuring. Let me translate this for you into plain English:

“Due to the fact that our senior management has totally screwed up the university’s finances, we need to decrease our staff costs by 20%. Since most staff would not accept to do the same work for 20% less pay, we will instead just fire 20% of the staff. If you are one of the 20% who are able to do your job and have been properly trained, we strongly advise you to immediately seek employment elsewhere. After all, you might find yourselves fired in the next round of cuts, if these ones don’t prove sufficient”.

The duties of the 20% who leave will be transferred to the 80% who remain. Since they will be required to carry out additional tasks beyond those that they currently undertake but were never trained to perform in the first place, it is natural that many of them will go on permanent sick leave or retire early, thus reducing our staff costs further and avoiding undue stress on persons we have failed to train properly.

As a result, academic staff currently engaged on less quantifiable activities such as research and teaching must shoulder some of the burden. We hope to avoid firing academic staff but be aware that you may also be terminated if performance targets are missed, especially if you are unable to undertake simple obligations to help the university to function properly instead of wasting all your energy on research and teaching.

The next phase of this process will be the outsourcing of many of the duties that the poorly trained 80% of staff and academics cannot do or are unwilling to do. We will invite tenders from different companies and always pick the lowest bidder, regardless of the quality of the services they are able to perform. However, be aware that academic staff who make use of these services must employ the companies contracted by the university and no others. You will also have to pay for these services from research grants or other income.

Spin‐off companies that you have created could also bid for some of these services, which might enable you to cover the costs during the 2–3 years before your fledgling company goes bankrupt.

Please also consider if and how you can outsource your own research and teaching, which would enable us to fire academic staff as well, in any future cost‐cutting exercise.

If this process is successful, the university hopes to recycle any excess savings into a new round of Strategic Interconvertibility and Sustainable Innovation (SISI) grants.

And above all, please do remember and implement our university’s new slogan “The only useful research is market research”.

*Kit from HR, aka Howy Jacobs*

